# Event-Based Surveillance at Community and Healthcare Facilities, Vietnam, 2016–2017

**DOI:** 10.3201/eid2409.171851

**Published:** 2018-09

**Authors:** Alexey Clara, Trang T. Do, Anh T.P. Dao, Phu D. Tran, Tan Q. Dang, Quang D. Tran, Nghia D. Ngu, Tu H. Ngo, Hung C. Phan, Thuy T.P. Nguyen, Anh T. Lai, Dung T. Nguyen, My K. Nguyen, Hieu T.M. Nguyen, Steven Becknell, Christina Bernadotte, Huyen T. Nguyen, Quoc C. Nguyen, Anthony W. Mounts, S. Arunmozhi Balajee

**Affiliations:** Centers for Disease Control and Prevention, Atlanta, Georgia, USA (A. Clara, T.T. Do, A.T.P. Dao, S. Becknell, A.W. Mounts, S.A. Balajee);; General Department of Preventive Medicine, Hanoi, Vietnam (P.D. Tran, T.Q. Dang, Q.D. Tran);; National Institute of Hygiene and Epidemiology, Hanoi (N.D. Ngu, T.H. Ngo);; Pasteur Institute, Ho Chi Minh City, Vietnam (H.C. Phan, T.T.P. Nguyen);; Preventive Medicine Center, Nam Dinh, Vietnam (A.T. Lai);; Preventive Medicine Center, Quang Ninh, Vietnam (D.T. Nguyen);; Preventive Medicine Center, An Giang, Vietnam (M.K. Nguyen);; Preventive Medicine Center, Ba Ria-Vung Tau, Vietnam (H.T.M. Nguyen);; Program for Appropriate Technology in Health, Seattle, Washington, USA (C. Bernadotte, H.T. Nguyen, Q.C. Nguyen)

**Keywords:** Public health surveillance, disease outbreaks, Vietnam, viruses, bacteria, International Health Regulations, global health security

## Abstract

Surveillance and outbreak reporting systems in Vietnam required improvements to function effectively as early warning and response systems. Accordingly, the Ministry of Health of Vietnam, in collaboration with the US Centers for Disease Control and Prevention, launched a pilot project in 2016 focusing on community and hospital event–based surveillance. The pilot was implemented in 4 of Vietnam’s 63 provinces. The pilot demonstrated that event-based surveillance resulted in early detection and reporting of outbreaks, improved collaboration between the healthcare facilities and preventive sectors of the ministry, and increased community participation in surveillance and reporting.

After several international outbreaks of infectious diseases, including severe acute respiratory syndrome in 2003, all World Health Organization (WHO) Member States, including Vietnam, agreed to comply with the revised International Health Regulations 2005 (IHR 2005) to ensure global health security (*1*). The IHR 2005 requires countries to develop early warning and response functions that can rapidly detect, report, and respond to—and thereby control—public health events. WHO defines early warning and response as “the organized mechanism to detect as early as possible any abnormal occurrence or any divergence from the usual or normally observed frequency of phenomena” (*2*). Two complementary types of surveillance form the foundation of a functional early warning and response: indicator-based surveillance (IBS) and event-based surveillance (EBS) (*2**,**3*).

In Vietnam, IBS is mandated by Circular 54, a Ministry of Health regulation, disseminated in 2015 (*4**,**5*). Circular 54 focuses primarily on reporting of case-based hospital admissions through an electronic system, the eCDS (electronic Communicable Disease Surveillance software). Several disease- or syndrome-specific sentinel surveillance programs complement eCDS, focusing on conditions such as dengue; hand, foot, and mouth disease; Japanese encephalitis virus; influenza-like illness; and severe acute respiratory infections.

WHO defines EBS as the organized collection, monitoring, assessment, and interpretation of mostly unstructured information from diverse ad hoc sources, including communities, schools, and media. Signals may represent unusual disease patterns that signify early signs of an outbreak or event (*2**,**6*). Both IBS and EBS generate signals, which might consist of reports of cases or deaths (individual or aggregated); potential human exposure to biological, chemical, or radiologic hazards; or occurrence of natural or human-made disasters. These signals, which are unfiltered reports, are first triaged and verified to confirm the occurrence of a true event that needs further investigation. Decision 134/QD-DP, issued in 2014 by Vietnam’s Ministry of Health’s General Department of Preventive Medicine (GDPM), describes national EBS procedures but is largely focused on signal identification through media scanning and omits collection of information from other sources, such as pharmacies, animal and agricultural sectors, community, workplaces, the private sector, and schools (*7*).

Regional Institutes in each of Vietnam’s 4 administrative health regions are responsible for implementing and overseeing surveillance and response. Within each region, Provincial Preventive Medicine Centers (PPMCs) lead these activities within their jurisdictions, involving the Regional Institutes for larger events. The PPMCs are supported by 2 lower administrative levels, the District Health Center (DHC) and Commune Health Station (CHS). The CHS is generally staffed by a medical professional and village health workers (VHWs), who are largely volunteers. The VHWs promote prenatal visits and vaccinations and in theory are responsible for reporting outbreaks from their communities. In addition, several community members called health collaborators assist VHWs in these tasks.

A 2014 assessment of Vietnam’s surveillance and reporting structures by a joint Ministry of Health and US Centers for Disease Control and Prevention (CDC) team found that the existing surveillance was largely IBS with reliance on healthcare facility (HCF) reporting that was case-based. HCFs were not required to report unusual patterns of unknown diseases, resulting in delays in detection of outbreaks and events caused by emerging pathogens (*5*). In addition, the team found that VHWs were underutilized and not actively engaged with detection and reporting of suspected outbreaks from their communities. Finally, the team found no alert thresholds established for routinely reported HCF data for many endemic seasonal diseases, such as dengue or hand, foot, and mouth disease.

To complement and reinforce the surveillance system, the GDPM in collaboration with CDC launched an EBS pilot project in 2016 focusing on communities and HCFs, including hospitals. Community EBS entailed reporting symptoms and unusual patterns that do not require specialized healthcare training from the communities by VHWs, health collaborators, and key informants. HCF EBS required healthcare workers to recognize and report unusual occurrences or disease patterns, such as a surge in admissions or healthcare worker sickness after patient exposure with similar illness.

For phase 1 implementation, GDPM selected the National Institute of Hygiene and Epidemiology and the Pasteur Institute of Ho Chi Minh City, the 2 larger Regional Institutes, and worked with them to select 2 pilot provinces per region. Criteria used to select provinces included support from the local government; availability of personnel for response; and previous occurrence of diseases of high concern, such as avian influenza. For phase 2, the intention was to pilot in 2 remaining Regional Institutes, including 2 provinces within their jurisdictions. Phase 1 of the pilot was implemented in 4 of Vietnam’s 63 provinces. We describe the steps of phase 1 implementation and its preliminary assessment results.

## Methods

### Establishing a Technical Working Group for EBS

The GDPM formed an EBS Technical Working Group (TWG) consisting of stakeholders from the Ministry of Health, including the 2 Regional Institutes, PATH (an international organization), CDC, WHO, and technical staff from the pilot province PPMC. In addition to guiding the EBS planning and preparations, the TWG served as the advisory group for implementation throughout the project. TWG members also served on an assessment team and later assisted in disseminating the assessment results to stakeholders.

EBS signals do not need to be disease specific. However, to reduce the background noise and to provide a framework for reporting, the TWG listed priority diseases and conditions that were important for early detection in Vietnam. Criteria for inclusion included diseases that 1) have large public health impact in the country, 2) are outbreak prone and pose a major public health threat, 3) have previously been prevalent and might reemerge, and 4) are slated for eradication or elimination. High-priority diseases identified were rabies, avian influenza, vaccine-preventable diseases, cholera, and emerging new diseases.

The TWG then drafted a list of signals that could serve as an early indication of the appearance of these priority diseases in the community. Community signals represented constellations of symptoms and patterns that do not require specialized healthcare training; signals aimed at HCF were based on unusual occurrences and/or disease patterns, such as surge in admissions.

The TWG drafted an Interim Technical Implementation Guideline and training materials (*8*). Other materials included posters and flyers to increase community awareness of the signals and need to report, notebooks for VHWs with printed signals and pages for notes, logbooks for recording signals, and a monitoring checklist for supervisory visits at each administrative level.

### Training the Public Health Workforce in EBS

A training of trainers workshop was conducted for the Regional Institutes and pilot provinces. These participants became master trainers and led cascade trainings in each province down to the commune level. At each level, a trainer from a higher administrative level provided mentorship and support.

### Resources for Implementation Support

In addition to external funding for training, each province received a one-time start-up grant for infrastructural improvements, including purchase of a limited number of computers for reporting, a one-time allowance for VHW cellular phone minutes, and the printing and distribution of logbooks and communication materials. During the pilot phase, EBS district and provincial focal points received a small monthly honorarium for EBS oversight and support.

### Enhancing Existing Information Flow and EBS Reporting

**Figure 1:**
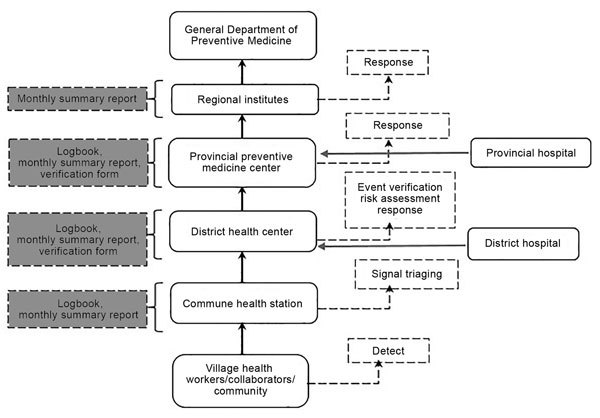
Existing surveillance and reporting system improved for event-based surveillance, Vietnam, September 2016–May 2017. Enhancements are shown in dashed boxes; the reporting tools at each level are shown in gray dashed boxes.

For EBS, the existing organizational structure and information flow from CHS to DHC to PPMC and to Regional Institutes was maintained with some enhancements (Figure 1), including 1) inclusion of VHWs at the CHS to identify and report signals; 2) addition of a triage step (the CHS decided which signals were “true” signals [rather than a spurious situation or nonthreatening rumor] before reporting these as events to DHCs); 3) training of DHCs and PPMCs in event verification and risk assessment; 4) distribution of logbooks for recording signals and events; 5) establishment of a requirement to immediately report events by phone call, in-person meeting, or email; and 6) training of healthcare providers to detect and immediately report signals to the correct public health unit.

### Assessing the EBS Pilot

Approximately 9 months after launch, the TWG assessed the EBS pilot, with qualitative and quantitative methods, for timeliness of detection and reporting of events, as well as EBS acceptability and sustainability at all levels. This assessment included 1) a retrospective data collection table sent electronically to all districts to collect logbook time stamps for event notification and response, 2) questionnaires sent electronically to all levels with acceptability and sustainability related questions, and 3) key informant interviews and focus group discussions through field visits.

We used 3 criteria to select field visit sites. First, we assessed districts that were performing optimally and suboptimally as defined by the metric signal incidence rate. Signal incidence rates were the number of signals detected from each district, adjusted by the district’s population and the number of days engaged in signal reporting. We defined optimal performance as districts with a signal incidence rate higher than the 50th percentile and suboptimal performance districts as districts with a signal incidence rate of the 50th percentile or lower. Second, we selected districts that investigated public health events reported through EBS that could be useful case studies. Third, we selected sites that were willing to receive assessors.

We sent the time stamp data extraction form to all 43 pilot districts. Approximately 7,000 participants encompassing EBS focal points and volunteers at all levels of the workforce in all 4 provinces received the acceptability/sustainability survey. In each province, 2 districts and 2 CHSs per district were selected for site visits and key informant interviews/focus group discussions deployment.

## Results

**Figure 2:**
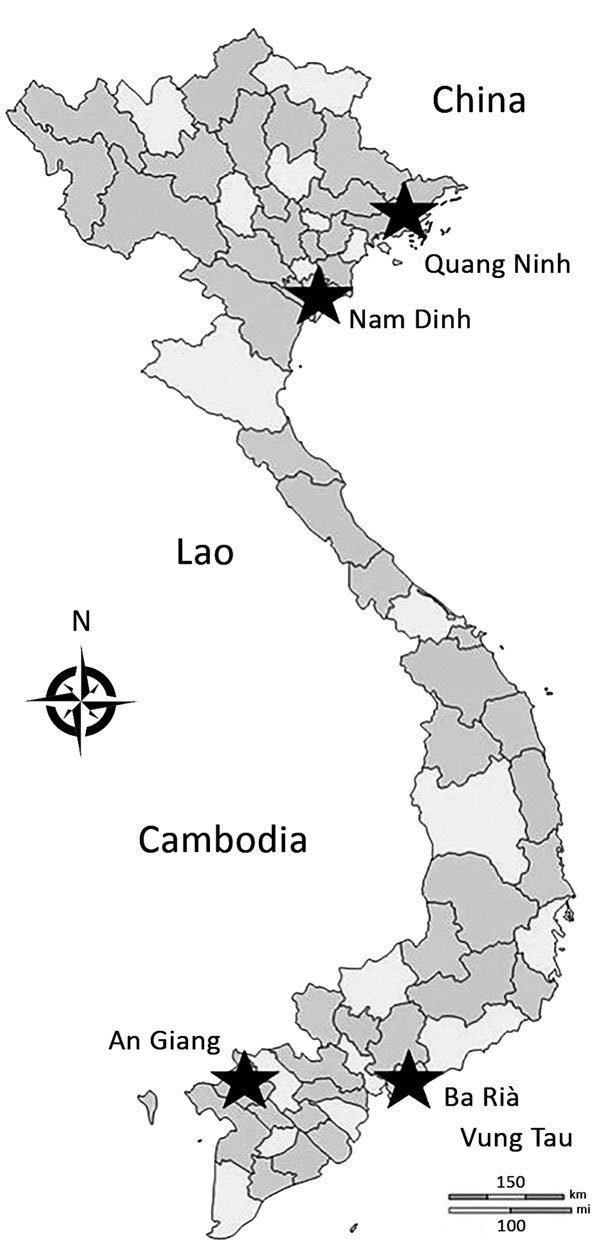
Provinces participating in event-based surveillance pilot project (stars), Vietnam, September 2016–May 2017.

The EBS pilot covered 7% of the total population of Vietnam (*9*). The provinces represented both rural and urban areas (Table 1; Figure 2).

**Table 1:** General characteristics of selected provinces in the pilot of event-based surveillance, Vietnam, September 2016–May 2017

Demographic and administrative profile	Province				
North			South	
Quang Ninh	Nam Dinh		Ba-Ria Vung Tau	An Giang
Demographics					
Population	1,211,300	1,850,600		1,072,600	2,158,300
Population density, persons/km^2^	198	1119		539	610
Urban population rate, %	62.5	18		50.1	31.1
No. households	316,732	555,605		256,336	524,759
Administrative division no.					
Cities under provinces	4	1		2	2
District-level towns	2	0		0	1
Rural districts	8	9		6	8
Wards	67	20		24	21
Commune-level towns (townlets)	8	15		7	16
Commune Health Station	111	194		51	119

### Resources and EBS Workforce

Twenty-four master trainers were trained in August 2016: two from each province and 16 GDPM and Regional Institute staff. A cascade training to lower administrative levels followed the master training. By October 2016, >7,000 persons in 4 provinces were trained to detect, record, and report signals and events, and 52 DHC staff were trained in basic risk assessment. Staff from every district, CHS, and public hospital within each province were trained, achieving 100% training coverage (Table 2).

**Table 2:** Number of persons trained in the pilot provinces, Vietnam, September 2016–May 2017*

Type of training	National level, GDPM	North				South			Total
RI, NIHE	Province		RI, PI-HCMC	Province	
	Quang Ninh	Nam Dinh		BRVT	An Giang
Training of trainers	4	6	2	2		6	2	2	24
Cascade									
Hospital	NA	NA	17	13		NA	8	14	52
District	NA	NA	42	30		NA	24	33	129
CHS	NA	NA	186	229		NA	82	156	653
VHWs/HCs	NA	NA	1,768	3,801		NA	710	888	7167
Total	4	6	2,015	4,075		6	826	1,093	8,025

*BRVT, Ba Ria-Vung Tau; CHS, Commune Health Station; GDPM, General Department of Preventive Medicine, Vietnam Ministry of Health; NIHE, National Institute of Hygiene and Epidemiology, Hanoi, Vietnam; PI-HCMC, Pasteur Institute, Ho Chi Minh City, Vietnam; RI, Regional Institute; VHWs/HCs, village health workers/health collaborators; NA, not applicable.

**Figure 3:**
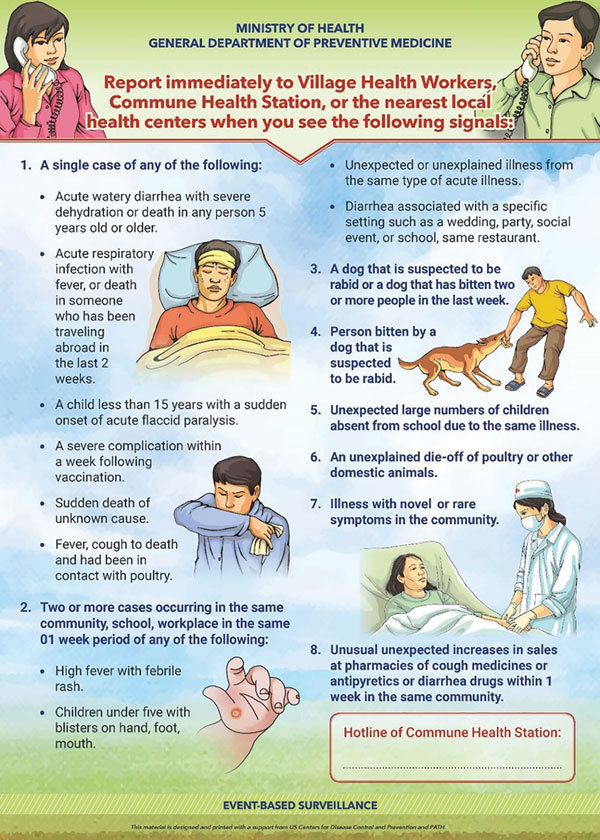
Poster displaying community-level signals for pilot of event-based surveillance, Vietnam, September 2016–May 2017.

At least 15,000 posters with community signals and reporting information were provided to CHSs (Figure 3). These posters were prominently displayed at public meeting places, CHS, village meetings, and other highly visible locations. In addition, 1,300 logbooks and 703,000 leaflets for the community were distributed (Table 3).

**Table 3:** Resources provided to implement event-based surveillance in pilot provinces, Vietnam, September 2016–May 2017*

Resource	Province					Total
North			South	
Quang Ninh, no.	Nam Dinh, no.		BRVT, no.	An Giang, no.
Computer + printer	15	11		9	12	47
Logbook						
For provincial level	2	2		2	2	8
For district level	26	22		16	22	86
For commune level	372	458		164	312	1,306
Communication materials						
Poster						
For community, displayed in public places	3,720	5,255		3,029	2,997	15,001
For HCFs at provincial level	60	40		100	60	260
For HCFs at district level	195	352		256	143	946
Other						
Leaflet for community	186,000	147,400		214,500	155,800	703,700
Plastic flyer holder	726	1,016		1,981	1,246	4,969
Handbook for VHWs/HCs	1,800	3,572		713	900	6,985

*BRVT, Ba Ria-Vung Tau; HCF, healthcare facility; VHW/HC, village health worker/health collaborator.

### EBS Pilot Assessment

**Figure 4:**
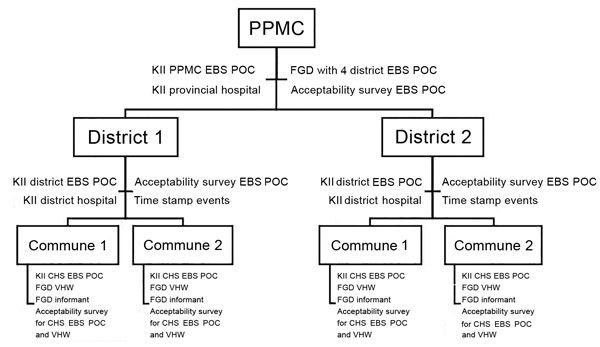
Assessment tools deployed at each site for assessment of an EBS pilot project, Vietnam, September 2016–May 2017. Acceptability survey and time stamp events tool were sent to all districts in the pilot provinces, not only to the sites selected for the FGD and KII site visits. CHS, commune health station; EBS, event-based surveillance; FGD, focus group discussion; FP, focal point; KII, key informant interview; POC, point of contact; PPMC, Provincial Preventive Medicine Center; VHW, village health worker.

As of July 1, 2017, we received 2,105 acceptability/sustainability surveys from 5 PPMC staff, 39 DHCs, 428 CHSs, and 1,633 VHWs. Twenty-four (56%) of 43 districts returned the timeliness data extraction forms. We conducted 34 key informant interviews, and 32 focus group discussions, both totaling 160 participants (Figure 4).

**Figure 5:**
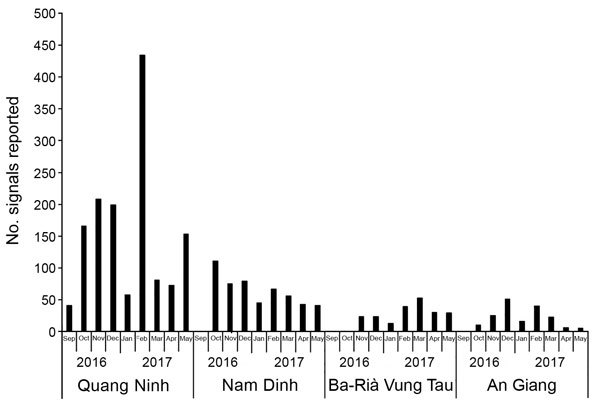
Number of signals reported to districts in the 4 event-based surveillance pilot provinces, Vietnam. Data were collected from the district monthly summary report during September 2016–May 2017.

During September 2016–May 2017, CHSs reported 2,520 signals to the districts (Figure 5). Quang Ninh province reported the largest number of signals. Of all 2,520 signals, 176 (7%) were verified as events by the districts and were responded to by the DHC or PPMC.

**Figure 6:**
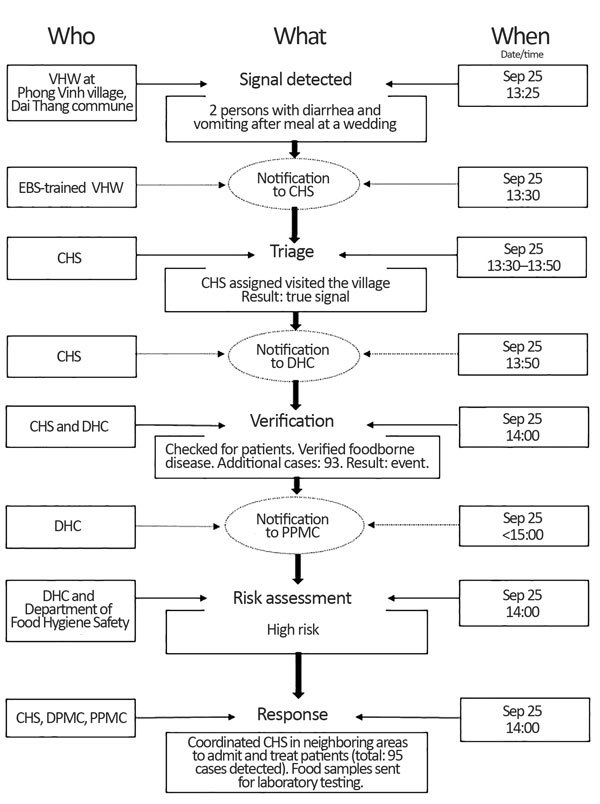
Case study of a cluster of food poisoning illustrating the value of EBS in early detection leading to rapid response, Dai Thang commune, Vu ban District, Nam Dinh Province, Vietnam, September 2016. CHS, Commune Health Station; DHC, District Health Center; DPMC, District Preventive Medicine Center; EBS, event-based surveillance; PPMC, Provincial Preventive Medicine Center; VHW, village health worker.

Although no preexisting timeline data were available for comparison, the pilot demonstrated that the mean times from detection to notification and detection to response were within 24 hours and 48 hours, respectively (Table 4) (*10*). We identified a case study illustrating the value of early event detection resulting in timely response (Figure 6). A trained VHW learned that diarrhea and vomiting developed in 2 persons who had attended a wedding party meal on September 25, 2016, at ≈13:00 hrs. The VHW called the CHS and reported the signal 30 minutes after learning of the episode. The CHS EBS focal point visited the village and, after confirming the signal, immediately reported to the DHC EBS focal point, who joined the CHS team. The investigation found 93 other affected persons, 38 of whom were hospitalized. The DHC reported the event to the PPMC, which conducted a risk assessment classifying the event as high risk and launched a response the same day. The time to notification to the DHC was within 30 minutes, and the time to response was within 3 hours.

**Table 4:** Time to notification and response during event-based surveillance pilot project, Vietnam, September 2016–May 2017

Type of event	No. events	Mean time to notification, h* (median [range])	Mean time to response, h† (median [range])
Suspected chickenpox	28	11 (12 [<1–24])	15.3 (12 [<1–48])
Hand, foot, and mouth disease	27	15 (12 [<1–171])	18 (12 [<1–171])
Suspected dengue	22	6.6 (2.5 [<1 −27])	36 (12 [5–318])
Avian influenza‡	14	3.4 (<1 [<1–12])	4.5 (1 [<1–15])
Foodborne disease	11	5 (<1 [<1 −24])	6.7 (<1 [<1–24])
Acute respiratory infection	10	9 (12 [1–12])	10 (12 [6–12])
Suspected mumps	9	9 (12 [<1–18])	18 (12 [<1–48])
Other	15	Not calculated	Not calculated
Total	136§	Not calculated	Not calculated

*Time from first detection to notification to the district level. †Time from first detection to response. ‡Avian influenza in poultry, not human cases. §From 176 events reported, 40 were excluded for timeliness analysis (incomplete, missing, incoherent or nonverified data).

**Figure 7:**
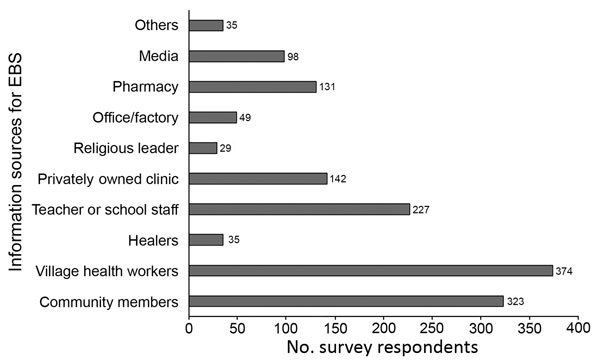
Sources contributing to signal detection and reporting through EBS at the community level in pilot provinces, Vietnam, September 2016–May 2017. Data were extracted from 428 acceptability survey questionnaires completed by Commune Health Station EBS focal points in July 2017. Each bar represents the number of survey respondents who identified the information source as contributing to EBS within the last 4 weeks. EBS, event-based surveillance.

At the community level, signals were being recognized and reported from multiple sources. The most frequent EBS reporters were VHWs, teachers, community members, traditional healers, veterinarians, and representatives from industrial complexes (Figure 7). Reported events included multiple suspected avian influenza poultry die-offs and human outbreaks of chickenpox, mumps, and foodborne disease.

During the key informant interviews and focus group discussions, interviewees reported that the signal language should be further simplified, including alternatives for medical terms such “severe,” “dehydration,” and “complications.” Furthermore, some of the guideline language was deemed overly academic and needed to reflect everyday language. Most interviewees appreciated the illustrations in the posters and leaflets and noted their usefulness in areas that included ethnic minority populations that did not read Vietnamese.

A total of 82%–88% of VHW, CHS, and district respondents reported that EBS is very important in detecting public health events and helps to detect public health events earlier than before (Table 5). In addition, ≈85% of VHW and CHS respondents and 77% of district respondents said they were willing to continue participating in EBS. Data collected during field visits substantiated these results (data not shown).

**Table 5:** Acceptability and sustainability of survey results, EBS pilot project, Vietnam, June–July 2017*

Indicator	VHW, %, n = 1,633	CHS, %, n = 428	DHC, %, n = 39
Agree that EBS is very important in the detection of public health events	87.2	87.6	82.1
Agree that EBS helps detect public health events earlier than before	87.1	88	84.7
Willing to continue taking part in EBS	85.2	84.1	77
Agree that EBS should be continued	85.4	82.2	79.5

*The 5 provincial-level staff who received the survey responded. All agreed that EBS is important and should be continued. CHS, commune health station, community level; DHC, district health center, district level; EBS, event-based surveillance; VHW, village health workers.

Key motivating factors for participation expressed by the VHWs were a sense of service to the community, opportunities to increase community ties, and improvement in community trust. Some VHWs also said that the EBS project better defined their responsibilities. Staff reported that the EBS project increased communications between different levels of the public health system, which aided in early detection of events and outbreaks.

## Discussion

The EBS pilot project builds on and expands the existing surveillance system in Vietnam to include community and HCF event-based surveillance. The pilot EBS implementation in Vietnam demonstrated earlier detection and reporting of outbreaks, improved collaboration among HCFs, the preventive health and animal health sectors of the government, and increased participation of communities in surveillance and reporting. Thus, EBS implementation contributes to Vietnam’s compliance with IHR 2005, thereby enhancing global health security.

The pilot initiative trained an existing network of VHWs and health collaborators to increase their awareness to look for and report signals as they appear in the community and to improve their understanding of patterns of disease that could signal the start of an outbreak. In most communes, the CHSs also recruited and trained additional community members as health collaborators through the current project. Most were persons with strong community ties, including money lenders, insurance agents, veterinary health staff, landlords, factory managers, community leaders, and others in a good position to directly observe community events. This wide participation broadened the sources of reporting and resulted in the reporting of numerous signals that otherwise would have been missed, such as school absenteeism reported by teachers and the resulting multiple detections of vaccine-preventable diseases (e.g., mumps and chickenpox). In contrast to reporting by clinicians from HCFs, VHWs recognized connections between cases in the community that doctors can miss, such as clusters among neighbors, co-workers, or persons with social connections.

The system did not rely on data reporting, aggregation, and analysis but rather used direct reporting methods to existing district and provincial authorities responsible for outbreak response. Based on the pilot implementation of EBS, it is plausible that focusing on patterns of occurrence in the community enabled outbreaks to be detected before they were large enough for HCFs to notice. Although all district and provincial public hospitals reported, no private hospitals and clinics participated in the EBS, making community-level participation critical to the detection process.

In the pilot districts, all events were detected and reported within 48 hours, and response was timely. Before EBS, such a rapid response by DHCs would not have been possible because ill persons would have to have been hospitalized to alert the system and, for certain diseases, traditional reporting often bypassed the CHSs. For example, foodborne illness events would first have to be reported to the Department of Food Safety and Hygiene, rather than the CHS, and ultimately to the DHC, resulting in delays. Similarly, animal events such as poultry die-offs or rabid dogs previously would have been reported to the Animal Health Department, and human health officials would not necessarily be alerted. During field visits, the DHC staff stated that because of the EBS pilot, multisectoral communication, such as between food safety and public health and human and animal health sectors, improved substantially.

The greatest challenge in quantifying EBS impact was lack of baseline outbreak data. Although Circular 54 requires outbreak reporting through eCDS, outbreak reports are not recorded even if detected, and therefore baseline data were not available. However, the absence of preexisting data demonstrates another important EBS contribution: the availability of data on outbreaks and events for planning public health interventions.

The assessment was an important part of the pilot and highlighted several problems that had to be rectified. Specifically, for some signals, wording needed to be simplified for VHWs, and the signal list itself needed to be more concise. In addition, for some diseases, such as hand, foot, and mouth disease, ongoing surveillance requires reporting of every case rather than clusters, creating some confusion. In some jurisdictions, leadership decided unilaterally to broaden signals to include single case reports, whereas the signal had been defined as a cluster, increasing the system’s sensitivity, but with a very low specificity. This change resulted in only 7% of all signals becoming public health events. In the future, adherence to accepted signal definitions by the workforce can be maintained with continuous training and experience. Based on the assessment, the guidelines and training materials were revised and will undergo pilot testing before scale-up (Table 6).

**Table 6:** Revised signals for community and healthcare facilities in provinces participating in event-based surveillance pilot project, Vietnam, June 2017

Facility type	Signal
Community	1 child <15 y of age with
	• Sudden weakness of limbs
	• Fever, rash, respiratory infection, and possibly red eyes
	A single case severe enough to require hospital admission or causing death of any of the following:
	• >3 rice watery stools in 24 h in any person >5 y of age with dehydration
	• A new respiratory infection with fever in a person who has traveled abroad in the past 14 d
	• A new respiratory infection with fever after contact with live poultry
	• Illness within 14 d after vaccination
	• Illness never seen before or rare symptoms in the community
	>2 hospitalized persons and/or death with similar type of symptoms occurring in the same community, school, or workplace in the same 7-d period
	Unexpected large numbers of
	• Children absent from school because of the same illness in the same 7-d period
	• Sales at pharmacies of many people buying medicines for the same kind of illness
	• People sick with the similar type of symptoms at the same time
	• Deaths of poultry or other domestic animals
	A dog that is suspected to be rabid or
	• A sick dog that has bitten someone
	• Any dog that has bitten >2 persons in the past 7 d
Healthcare facility	Severe illness requiring hospital admission in healthcare workers after they cared for patients with similar symptoms
	>2 cases of severe acute respiratory infections within 7 d in the same community or household
	Large unexpected, sudden increases in admissions for any illness of the same type, including patients in intensive care units
	Severe, unusual, unexplainable illness, including failure to respond to standard treatment

Another challenge was the number of respondents to the online survey. The online acceptability survey was sent to the entire EBS workforce in the pilot provinces, but GDPM closed the survey after only 3 weeks. Thus, only a relatively small proportion (25%) of VHWs respondents were able to complete the survey, which might have limited the representativeness of some of the survey findings.

Despite the above limitations, experience gained through the pilot project in Vietnam might be useful for other countries looking to launch EBS. To that end, we recommend the following:

1. Early in the implementation process, form a TWG led and coordinated by the Ministry of Health and with participation from all stakeholders. A TWG facilitates coordination of technical and financial resources and a better understanding of the existing landscape of systems and actors, thereby reducing redundancies and improving buy-in from implementers.
2. Position EBS to fit within the existing legal framework for surveillance and reporting. The EBS TWG for this project researched the existing regulations around reporting and demonstrated how the program complemented the existing systems rather than something additional. The TWG also avoided introduction of new technologies and regulations whenever possible to minimize disruption.
3. Include focused training on risk assessment to help staff to prioritize events for investigation.
4. Provide repeated follow-up refresher training.
5. Build in resources for supportive monitoring visits and mentoring of district-level staff and below and include an evaluation process.
6. Engage community leaders early in the process to ensure uptake of the program.
7. Design pilot projects that can be scaled up.

Based on the experience gained by the initial EBS pilot project, the Vietnam Ministry of Health expanded the pilot to 2 new provinces in the central and highlands areas. The TWG revised training materials based on the findings of a final assessment and drafted with GDPM a decision letter to formally integrate EBS into the national surveillance system. The vice minister of health issued a mandate in March 2018 that directed all provinces to integrate event-based surveillance into the national surveillance strategy, ensuring sustainability of the CEBS program. The formalization of EBS as a Ministry of Health regulation will enable the provinces to seek funds in the provincial budget to support EBS. With the Ministry of Health mandate, revised EBS materials, and experience gained by launching an EBS pilot, Vietnam’s surveillance system will soon function as an effective early warning and response system.
